# The power of many: The role of global matching in the episodic flanker compatibility effect

**DOI:** 10.3758/s13421-025-01733-w

**Published:** 2025-05-30

**Authors:** Gordon D. Logan, Simon D. Lilburn

**Affiliations:** Vanderbilt University, Nashville, TN USA

**Keywords:** Compatibility effects, Focused attention, Memory retrieval, Flanker task, Attention and memory

## Abstract

The episodic flanker task is a memory analog of the classic perceptual flanker task. It was designed to test the conjecture that memory retrieval is perceptual attention turned inward. It measures the sharpness of the focus of attention on memory and produces episodic compatibility effects from flanking items analogous to the perceptual flanker task. Here we ask whether the episodic flanker compatibility effect results from a *local match* between the probe item and the cued item in the memory list, a *global match* between the entire (multiletter) probe and the memory list, or a combination of the two. We report two episodic flanker experiments that manipulate the compatibility of near (adjacent to the target) and far (nonadjacent) flankers independently. Local matching predicts no effect of remote targets. Global matching predicts that remote flankers will modulate the compatibility effect, reducing it when one is compatible and the other is incompatible. The results of both experiments confirmed the global matching prediction. A third experiment manipulated near and far flankers in a classic perceptual flanker task and found that far flankers modulated the compatibility effect in the same way, strengthening the parallels between episodic and perceptual flanker tasks. We conclude that the episodic flanker compatibility effect, like the perceptual effect, depends on both local and global matching. Our results provide converging evidence for the idea that memory retrieval is perceptual attention turned inward.

**Supplementary Information:**

The online version contains supplementary material available at 10.3758/s13421-025-01733-w.

## Introduction

Since William James (1890), psychologists have investigated the close relations between attention and memory (Broadbent, 1957; Chun et al., 2011; Cowan, 1988; Craik & Tulving, 1975; James, 1890; Nobre et al., 2004; Norman, 1968; Rugg et al., 2008; Woodman et al., 2022). We have been exploring the conjecture that they may be one and the same: The same attentional processes operate on representations in perception and memory. Memory retrieval is perceptual attention turned inward; perceptual attention is memory retrieval turned outward (Logan et al., 2021, 2023b; Logan, Afu, et el., 2024; Logan et al., 2024a, 2024b). Our exploration is based on the idea that attention and memory must solve the same computational problem: searching a database to find desired information. Memory records perceptual experience, so the databases have similar content and similar structure, which constrain the search process in similar ways. Empirically, this implies strong parallels between perceptual attention tasks and memory tasks performed on similar structures. For example, serial recall of a list from memory requires the same iterative attention switching from one item to the next as serial report from a perceptual display (Logan, 2021). More broadly, the same manipulations of content and structure should produce similar effects in perceptual attention and memory retrieval.

Theoretically, the conjecture that memory retrieval is attention turned inward implies that models of perceptual attention can be applied to memory retrieval and models of memory retrieval can be applied to perceptual attention. Models of attention make explicit assumptions about how a “spotlight” is focused on one item among many; models of memory make explicit assumptions about how a retrieval cue selects one item out of many. There are clear parallels between the formal models, which suggest that a single model of selection may account for attention turned inward and outward. The retrieval cues in memory theories may be viewed as spotlights of attention turned inward that produce the same effects as spotlights turned outward. This view is the heart of our conjecture. It provides an economy of mechanism and integrates disparate fields of research theoretically and empirically.

We have tested the conjecture with an *episodic flanker task* (Logan et al., 2021), which adapts the famous B. A. Eriksen and Eriksen (1974) perceptual flanker task to memory retrieval. It produces compatibility effects and measures of the sharpness of the focus of attention that are directly analogous to the effects in the perceptual flanker task, which can be understood with the same models. Experiments 1 and 2 distinguish global and local contributions to the episodic flanker compatibility effect, testing hypotheses derived from theories of the perceptual compatibility effect. Experiment 3 applies the same tests to the perceptual flanker task.

**Fig. 1:**
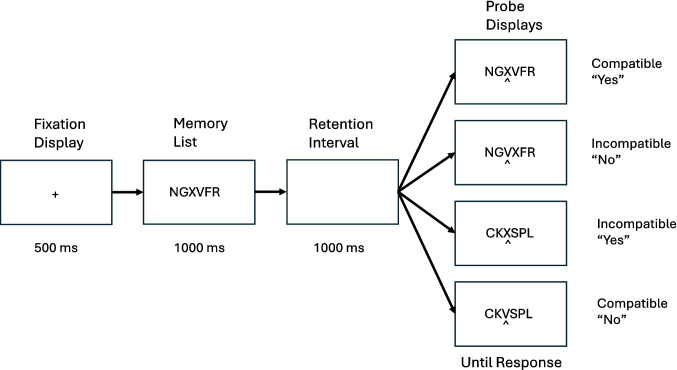
Events on a trial in the episodic flanker task. Each trial began with a fixation display exposed for 500 ms, followed by the memory list for 1,000 ms, followed by a 1,000-ms retention interval with a blank screen, and then a probe display containing six letters with a caret (^) cue underneath one of the letters, which was exposed until the subject responded. Compatibility is defined in terms of the relation between the response required to the cued letter and the response elicited by the letters that flank the cued letter. In “same” probes (top two), the flanking letters are the same as the list and are presented in the same order. “Same” probes point to a “yes” response and are compatible with cued letters that require a “yes” response and incompatible with cued letters that require a “no” response. “Different” probes point to a “no” response and are incompatible with cued letters that require a “yes” response and compatible with cued letters that require a “no” response

The episodic flanker task is illustrated in Figu. 1. Subjects are presented with a memory list followed after a brief retention interval by a probe list in which one of the items is cued with a caret (^) underneath it. The task is to say whether the cued item occurred in the same position in the memory list. The lures are selected from other positions in the list, so successful performance requires focusing on the cued item and ignoring distractors that surround it, like the perceptual flanker task. The compatibility effect is produced by manipulating the nature of the flankers in the uncued positions in the probe. They are either the *same* as the memory list (top two probes in Fig. 1) or *different* from it (bottom two probes). *Same* flankers match the memory list and activate a “yes” response. They are compatible with cued items that match the list and require a “yes” response (top probe). They are incompatible with cued items that mismatch the list and require a “no” response (second probe). *Different* flankers mismatch the memory list and activate a “no” response. They are incompatible with “yes” responses (third probe) and compatible with “no” responses (fourth probe). Like the perceptual flanker compatibility effect, response time (RT) and error rate are lower for compatible responses (same “yes,” different “no”) than for incompatible responses (different “yes,” same “no”).

We have observed robust episodic flanker compatibility effects in RT and error rate in 11 experiments (Logan et al., 2021, 2023b; Logan, Afu, et el., 2024; Logan et al., 2024a, 2024b). They occur with a broad range of materials, including letters, words, colors, and pictures (Logan et al., 2024a, 2024b). They are unaffected by preparation interval, which suggests they reflect processing after attention is focused on the cued position in the list rather than the processing that orients attention to the cued position (Logan et al., 2023b). The compatibility effect is *episodic* because it is produced by unique stimuli that appear just once in a specific place and time (Tulving, 1972).

The conjecture that memory retrieval is attention turned inward goes deeper than parallel empirical effects. We applied three formal models of serial memory that implement three different approaches to attention (space-based, object-based, and template-based) to the episodic flanker task, interpreting their retrieval cues as spotlights of attention turned on the memory representations. Each model predicts episodic flanker compatibility effects analogous to perceptual flanker compatibility effects and provides measures of the sharpness of the focus of attention on memory. The three models fit well and accounted for the observed effects, supporting our conjecture (Logan et al., 2021). The fits were not very different, which is bad news from the usual perspective of finding the best model and discarding the others but good news from the perspective of making general predictions (Anderson, 1978).

In this article, we test the conjecture further by investigating the contributions of local and global foci of attention to the episodic flanker compatibility effect, inspired by models of the perceptual flanker effect that assume global and local foci (Cohen et al., 1992; Hübner et al., 2010; Ulrich et al., 2015; White et al., 2011). We distinguish between a *local match* process that focuses on the cued location and samples information from the cued item and its immediate neighbors and a *global match* process that focuses on the whole list and samples information from all the items (Logan et al., 2021). The local match is necessary to respond accurately because lures come from different positions in the list. The global match cannot support accurate responding because flanker identity (same, different) is an invalid cue for “yes” and “no” responses. The flankers agree with the cued letter half the time (suggesting the same response) and disagree with it half the time (suggesting opposite responses). Responding to the global match by itself without considering the local match would produce an error rate of 50%.

Logan et al. (2021) implemented local and global matches in the three models, using their retrieval cues as spotlights of attention that activate the cued item and its neighbors in proportion to their similarity to the cued position. In each model, similarity *s*(*i,j*) of two positions *i* and *j* is (approximately) an exponential function of distance between positions in the list:

1 $$s\left(i,j\right)={\rho }^{|i-j|},$$

where *ρ* ranges from 0 to 1. (It is exactly exponential in one model and approximately exponential in the other two.) The *ρ* parameter determines the sharpness of the focus of attention on the cued position. The lower the value, the sharper the focus, the smaller the contribution from adjacent items. Items are represented as (orthogonal “one-hot”) vectors ***η***_*I*_ and the retrieved information about each item is the product of the similarities and the item vectors: *s*(*I,j*) × ***η***_*I*_. The local match (***m***_*j*_ when position *j* is cued) is computed by summing the activated item vectors:

2 $${{\varvec{m}}}_{{\varvec{j}}}=\sum_{i=1}^{N}s\left(i,j\right)\times {{\varvec{\eta}}}_{{\varvec{i}}}.$$

A representation of the cued item in the probe is constructed in the same way, producing a vector ***p***_*j*_. The decision is driven by the similarity between ***m***_*j*_ and ***p***_*j*_, expressed as the dot product between the vectors.

All items in the list contribute to the local match, weighted by the similarity of their positions to the cued position. The item in the cued position contributes the most (*s*(*I,i*) = 1) and adjacent items contribute more than remote items (i,e, *s*(*i,j*) = *ρ* for items ± 1 position away from the cued position, *s*(*i,j*) = *ρ*^2^ for items ± 2 positions away, and so on). The dominance of information from the cued position ensures the accuracy of the local match. The contributions of the flankers are responsible for the compatibility effect in the local match. When they are compatible, the flankers and the cued item support the same response. When they are incompatible, they support the opposite response.

Logan et al. (2021) modeled the global match ***M***_*G*_ by calculating the local match at each position and summing the match vectors over position:

3 $${{\varvec{M}}}_{G}=\sum_{i=1}^{N}{{\varvec{m}}}_{i}.$$

The decision is driven by a weighted combination of local and global matches:

4 $${drift}_{i}=q\times {{\varvec{m}}}_{i}+\left(1-q\right){\times {\varvec{M}}}_{G}.$$

Equation 4 shows how the global match contributes to the compatibility effect. Its contribution is maximal when the flankers are the same as the memory list and minimal when the flankers are different. Thus, same flankers will contribute to a “yes” response, facilitating “yes” responses to the cued item and impairing “no” responses. Different flankers will have the opposite effect. They contribute to a “no” response, impairing “yes” responses and facilitating “no” responses to the cued item.

Logan et al. (2021) compared versions of the three models that included both the global match and the local match with models that included only the local match (global weight (1 − *q*) = 0). They found that the combination of local and global matches provided the best account of the compatibility effect and other aspects of the data.

We have used experimental manipulations to examine the contributions of global and local matches to the compatibility effect empirically. Logan et al. (2021) manipulated the similarity between the probes and the memory list. One experiment compared same and different contexts with *scrambled* contexts, which presented the letters from the list in a different random order (list: ABCDEF, probe: EACBFD). Another experiment compared probes that differed from the list by *swapping* one or two pairs of items (list: ABCDEF, swap 1 probe: AECDBF, swap 2 probe: AECFBD). Compatibility contrasts were smaller for similar contexts than for same contexts in both experiments, consistent with global matches that align each letter in the probe with each letter in the memory list.

Logan et al., (2024a, 2024b) examined the role of global matches by asking whether a single distractor adjacent to the target can produce an episodic flanker compatibility effect. We found it can. The compatibility effects were about as large as those observed in previous multiple-flanker experiments for RT but smaller than the previous experiments for error rate (see Logan et al., 2024a, 2024b, Table 4). A second experiment manipulated the distance between the single flanker and the cued position in the probe display (list: ABCDEF, near flanker: ##CD##, far flanker A#C###). We found the compatibility effect in RT only when the flankers were immediately adjacent (near) to the targets. The compatibility effect in error rate was numerically larger for near than for far flankers, but the difference was not significant. Together, the two experiments suggest that near flankers (and thus local matches) are sufficient to produce the episodic flanker compatibility effect. They raise the possibility that remote flankers (and thus global matches) do not contribute to the effect. The Logan et al. (2021) data with scrambled and swapped flankers are ambiguous because scrambling and swapping affected both near and far flankers.

Experiments 1 and 2 addressed the role of global matching in the episodic flanker compatibility effect by manipulating the compatibility of near and far distractors directly and independently. There were four conditions: *Old-Old* (*OO*), in which both near and far flankers are old (from the memory list, e.g., list: ABCDEF, probe: ABCDEF), *New-New* (*NN*), in which both near and far flankers are new (not from the memory list, e.g., list: ABCDEF, probe: SBTRSV), *Old-New* (*ON*), in which the near flankers are old and the far flankers are new (e.g., list: ABCDEF, probe: ABCRSV), and *New-Old* (*NO*), in which near flankers are new and the far flankers are old (e.g., list: ABCDEF, probe: SBTDEF). The flanker compatibility effect from OO and NN conditions replicates the conditions in previous experiments, where flankers were all old or all new. Near and far flankers are associated with the same response. The flanker compatibility effect from ON and NO conditions pits near flankers against far flankers. Global matching assumes that near and far flankers both contribute to the compatibility effect, so it predicts that the effect should be stronger in the OO and NN conditions, where near and far flankers are associated with the same response, than in the ON and NO conditions, where near and far flankers are associated with opposite responses. Local matching assumes that only near flankers contribute to the compatibility effect, so it predicts that the effect in the ON and NO conditions should be the same as the effect in OO and NN conditions because the near flankers are the same in the OO and ON and in the NN and NO conditions. We tested these predictions in Experiments 1 and 2.

Both experiments presented lists of six consonants arranged in a horizontal row, followed by a brief retention interval, and a probe containing six consonants, as in our previous episodic flanker experiments (Logan et al., 2021, 2023b; Logan et al., 2024a, 2024b; see Fig. 1). A caret cue (^) was presented under one of the letters and the task was to indicate whether the cued letter in the probe was the same as the letter in the cued position in the memory list. All six positions were cued, so subjects had to remember the whole list. On “yes” trials, the letter in the cued position matched the letter in that position in the list. On “no” trials, lures were presented in the cued position in the probe. The lures were letters one position to the left or right of the cued position in the memory list, so subjects had to focus sharply on the cued position.

The two experiments implemented the ON-NO manipulation in different ways. In Experiment 1, old and new items were presented in the left or right sides of the probe (list: ABCDEF, ON probe: ABCRST, NO probe: QBVDEF). In Experiment 2, old and new items were centered on the cued position, so the target always had adjacent flankers of the same type (both old or both new) no matter which position was cued (list: ABCDEF, ON probe QBCDST, NO probe: AQCSEF). In both experiments, the local matching hypothesis predicts the same compatibility effects for ON and NO probes as for OO and NN probes. The global matching hypothesis predicts smaller compatibility effects for ON and NO probes than for OO and NN probes.

## Experiment 1

The first experiment implemented the ON-NO manipulation by presenting probes with three old items to the left or right of three new items, so the left side of the probe was old and the right side new or vice versa. This arrangement allowed us to assess the effects of serial order in retrieval (cf. Mewhort et al., 1969). Episodic flanker experiments show consistent serial position effects in RT. It increases linearly for Positions 1–3, levels off for Positions 4 and 5, and decreases for Position 6 (Logan et al., 2024a, 2024b, 2021, 2023b; Logan et al., 2024a, 2024b). We have interpreted the serial position effect as the time taken to orient attention to the cued position and assumed that the orienting process serially scans the memory list from left to right or from the ends inward (Fischer-Baum & McCloskey, 2015; Logan et al., 2023a). From this perspective, we might expect stronger compatibility effects for ON and NO probes on the left side of the display because subjects never orient to contradictory flankers. Compatibility effects on the right side of the display may be reduced because subjects first orient to positions with flankers that contradict the flankers around the cued position.

### Method

**Subjects** We tested 32 subjects recruited online through Prolific (https://www.prolific.co/). With this sample size, we found robust compatibility effects in 11 previous experiments (Logan et al., 2024a, 2024b, 2021, 2023b). We included only subjects 18–40 years of age, located in the USA, with English as their first language, with an approval rating of at least 95, who performed with at least 60% accuracy on the episodic flanker task. We replaced seven subjects who failed to meet the accuracy criterion. The experiment involved a single 1.5-h session. Subjects were paid US $12 per hour. The study was approved by the Vanderbilt University Institutional Review Board.

Subjects reported their age and gender. The mean age of the subjects was 30.84 years (*SD* = 5.73 years). The gender distribution (male:female:prefer-not-to-say) was 16:16:0.

#### Apparatus and stimuli

The experiments were conducted online on subjects’ personal computers. Subjects were instructed to use Google Chrome or Mozilla Firefox to complete the experiment. Phone and tablet users were excluded in the Prolific intake, and the experiment would not run on their browsers. The trials for each session were generated individually and sent to subjects’ computers using a custom Python backend. The experiment was controlled by JavaScript in the web browser using a custom function written to operate in jsPsych (de Leeuw, 2015). When the experiment started, subjects’ web browsers were instructed to enter full-screen mode to reduce distraction.

The memory lists were random lists of six consonants sampled from a set of 20 (excluding vowels and *Y*). They were presented as capital letters in Courier font in a horizontal line centered on the fixation point. The background of this display, and all the others, was set to mid-gray ([127, 127, 127] in 24-bit RGB values). The probe displays were lists of six letters appearing in the same positions as the memory list. The probe displays presented a probe item in a cued position, indicated with a caret (^) presented underneath it. The other positions were filled with consonants. Each letter was 100 pixels high and 60 pixels wide. The letters were spaced 120 pixels center to center. Memory lists and probes were 660 pixels wide.

#### Procedure

Each trial began with a fixation cross presented in the center of the screen for 1,000 ms. Then the memory list was presented for 1,000 ms, followed by a blank screen for 1,000 ms, and then a probe display containing an item from the display (at lag − 1, 0, 1) and five flanking letters was presented. Subjects were required to decide whether the probe item appeared in the cued position in the memory list, pressing M (or Z, counterbalanced) to indicate “yes” and Z (or M) to indicate “no.” Lag 0 trials required a “yes” response and Lag ± 1 trials required a “no” response. After subjects’ response, the screen went blank for a 1,000 ms intertrial interval. There were 768 trials. Breaks were given every 96 trials.

The basic design required 24 trials to balance lag and cue position: four trials representing lag (two for Lag 0 and one each for Lags ± 1) for each of the six cue positions. Lag − 1 was not possible for cue Position 1 and Lag + 1 was not possible for cue Position 6. These lags were “reflected,” so there were two Lag + 1 trials for Position 1 and two Lag − 1 trials for Position 6 to complete the design. Each context condition (OO, NN, ON, NO) included eight replications of the basic design, yielding 192 trials per condition and 768 trials in total. Context conditions, cued positions, and lags appeared in random order. A separate random order was prepared for each subject.

The instructions were written and presented using a self-paced series of manually controlled slides. Subjects were allowed to review the instructions if they wished. At the end of each block, a screen was presented indicating the overall accuracy for the preceding block, and subjects were allowed to take a self-timed break. Every 5 min, the experiment checked whether accuracy was greater than 60%. If subjects fell below this criterion, they were warned to improve performance and given an opportunity to review the instructions. On the third warning, subjects were excluded from the experiment but were paid nevertheless.

#### Data analysis

The purpose of this experiment is to determine whether the compatibility effect in the episodic flanker task varies with the compatibility of near and far flankers. We addressed these questions with planned linear contrasts of the form ${\sum }_{i=1}^{N}{c}_{i}{M}_{i}$ such that ${\sum }_{i=1}^{N}{c}_{i}=0$, where *N* is the number of conditions, *c*_*i*_ are the weights on means 1 to *N*, and *M*_*i*_ are the means. We calculated each contrast separately for each subject and then did a *t* test comparing the mean value to 0 using the standard error of the mean as the error term specific to each contrast. We describe each contrast as a set of weights and the set of conditions they apply to. We assessed the compatibility effect in RT from correct trials and error rate for all trials in OO-NN and ON-NO contexts (separately) with a contrast using weights {− 1 + 1 + 1 − 1} for conditions {same-yes same-no different-yes different-no}. We asked whether compatibility effects differed between OO-NN and ON-NO contexts by comparing their contrast values with another contrast with weights − 1 for the OO-NN contrast and + 1 for the ON-NO contrast. We calculated the contrasts separately for probes on the left (Positions 1, 2, 3) and right (Positions 4, 5, 6) sides of the display. These contrasts focus directly on the theoretically relevant questions. Our analyses were planned to collapse over serial position to maximize the number of observations per cell of the design (96 per subject). We report an analysis of serial position in the Appendix.

The raw data and the means for each subject in each condition of each experiment are available on the Open Science Framework (https://osf.io/sv8ag/) for readers interested in other analyses. Because the effects of interest are planned contrasts that compare conditions to assess patterns in the data, we could not construct error bars for the contrasts around the individual mean RTs and error rates that would permit statistical inferences about the pattern. The relevant standard error is the standard error of the mean contrast, which reflects the variability in the pattern (i.e., the variability of differences between conditions) and not the variability in individual mean RTs and error rates in each condition that contributes to the contrast. Consequently, there are no error bars in our figures.

## Results and discussion

**Fig. 2:**
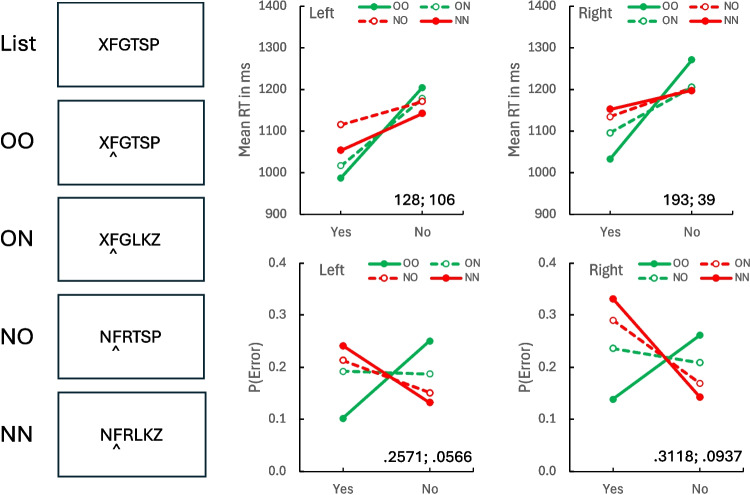
Episodic flanker task list and probes (left column), mean response time (RT; middle and right top panels), and error rate (P(Error); middle and right bottom panels) for left side cues addressing Positions 1–3 (left panels) and right side cues addressing Positions 4–6 (right panels). OO = old, old (both near and far contexts old); NN = new, new (both near and far contexts new); ON = old, new (near context old, far context new); NO = new, old (near context new, far context old). The values of the compatibility contrasts for OO-NN and ON-NO probes (respectively) are inset in each panel. (Color figure online)

Mean RTs (top) and error rates (bottom) are plotted as a function of response (“yes” vs. “no”) and context condition (OO-NN, ON-NO) in the middle and right columns of Fig. 2. The middle column shows compatibility effects when the left side was cued (Positions 1, 2, 3). The right column shows compatibility effects when the right side was cued (Positions 4, 5, 6). The values of the OO-NN and ON-NO compatibility contrasts are inset in each panel.

The data in the OO-NN conditions show compatibility effects in RT and error rate for probes on the left and the right, replicating the previous experiments that used OO and NN probes. The data in the ON-NO conditions show compatibility effects that are generally smaller than the OO-NN effects. They were consistently smaller for error rate. For RT, the ON-NO effects were smaller for probes on the right side of the display and about the same for probes on the left side of the display. The RTs from the right side and the error rates from both sides show strong effects of remote flankers, consistent with global matching. The RT results from the left side are more consistent with local matching.

These conclusions were supported by inferential statistics. Contrasts assessing the compatibility effect were significant for RT and error rate for OO-NN probes on both sides (see Table 1). The data in the ON-NO conditions show weaker compatibility effects. The compatibility contrasts for ON-NO were significant for RT on the left but not on the right side. The compatibility contrasts for ON-NO were significant for error rate on both sides. A contrast comparing OO-NN and ON-NO compatibility effects on the left side was not significant for RT, suggesting a local match. However, the OO-NN versus ON-NO contrast was significant for error rate, suggesting a global match. The contrast comparing OO-NN and ON-NO compatibility effects on the right side were significant for both RT and error rate, consistent with a global match.

**Table 1:** Contrasts evaluating the episodic compatibility effect in response time and error rate in Experiment 1

	*t*(31)	*p*	*SEM*	*N* > 0	*BF*_*10*_
*Response time*					
Left OO-NN	6.1433	<.0001	20.8155	28	21,149.48
Left ON-NO	4.9130	<.0001	21.5054	27	826.48
Left OO-NN v ON-NO	1.0870	.2054	20.4408	18	0.3242
Right OO-NN	7.1588	<.0001	27.0208	22	298,732.4
Right ON-NO	1.4793	.1492	26.6596	22	0.5051
Right OO-NN v ON-NO	3.6432	.0010	42.2711	25	33.3266
*Error rate*					
Left OO-NN	6.5693	<.0001	.0391	31	64,706.46
Left ON-NO	2.4445	.0204	.0232	21	2.4231
Left OO-NN v ON-NO	4.4172	.0001	.0454	28	228.85
Right OO-NN	7.4777	<.0001	.0417	29	675,709.2
Right ON-NO	2.9435	.0061	.0318	24	6.7194
Right OO-NN v ON-NO	3.5891	.0011	.0608	22	29.2786

*OO* old near and far contexts, *NN* new near and far contexts, *ON* old near and new far contexts, *NO* new near and old far contexts, *SEM* standard error of the mean, *N* > 0 number of subjects showing a contrast greater than zero, *BF*_*10*_ Bayes factor supporting the alternative hypothesis

Overall, the data are consistent with a mixture of local matching and global matching. Local matching accounts for accuracy being well above chance (hit − false alarm rate = 0.5945 across all conditions). Global matching accounts for the reduction in the compatibility effect from OO-NN to ON-NO probes in RT and error rate for right-side probes and in error rate for left-side probes. However, local matching accounts for the equivalent compatibility effects in RT for the OO-NN to ON-NO contexts for left-side probes. This latter result may be consistent with the interaction between left-to-right orienting and context we described in the introduction to this experiment: If subjects orient to the cued position by scanning left to right, they will not encounter contradictory contexts on their way to the cued position with left-side cues, but they will scan past three contradictory contexts on their way to the cued position with right-side cues. Experiencing contradictory contexts may reduce the compatibility effect, as it mixes support for both responses. RT was 53 ms longer and error rate was 0.0387 larger on the right side than on the left, suggesting left-to-right orienting, but neither difference was significant, *t*(31) = 1.9829, *p* = 0.0563, *SEM* = 27, BF_10_ = 1.0622, *N* > 0 = 17 for RT; *t*(31) = 1.9916 *p* = 0.0553, *SEM* = 0.0194, BF_10_ = 1.0776, *N* > 0 = 21 for error rate. That is not strong evidence of left-to-right orienting.

Another possibility is that global matching has a left-side bias, so that flankers on the left have a stronger influence than flankers on the right. If so, the rightmost flankers in the ON condition might not contribute much to the global match, allowing it to mimic a local match on the left side of the display. Many theories of serial memory assume a primacy bias in which cuing the list activates items in decreasing proportion to their distance from the beginning of the list (Brown et al., 2007; Henson, 1998; Lewandowsky & Farrell, 2008; Logan & Cox, 2021). If the global match uses such cuing to access the list, it may inherit the primacy bias and represent items on the left more strongly than items on the right. Of course, this is speculation. Understanding the nature of global matching is an important goal for future research.

## Experiment 2

Presenting old and new items on the left and right sides of the probe displays in Experiment 1 allowed us to test hypotheses about global and local matches and assess the effects of left and right positions in the list. However, that manipulation created some differences in the flanking items between probe positions. For left side ON probes, the nearest new flanker is two positions away when Position 1 is cued, one position away when Position 2 is cued, and adjacent to a new flanker when Position 3 is cued. The same reasoning applies to right side probes in Positions 6, 5, and 4. These mixtures of distances to different flankers may compromise the comparison of OO-NN and ON-NO contexts. The cued item in Position 1 has two new or two old flankers next to it, making global and local matching harder to distinguish. The cued item in Position 3 has an old flanker on one side and a new flanker on the other side, pushing the local match in different directions and reducing the difference between OO-NN and ON-NO compatibility effects, producing a local effect that mimics the global effect. The cued item in Position 2 is optimal, as it presents one flanker of the same type on each side of the target to support the local match.

Experiment 2 was designed to implement this optimal design. We cued all six positions in the probe. The flankers immediately adjacent to the cued position were always the same type, both old or both new. The nearest flanker of a different type was always two positions away from the target. The predictions were the same as in Experiment 1: Local matching predicts no difference between OO-NN and ON-NO compatibility effects because only the adjacent flankers intrude in the spotlight. Global matching predicts smaller compatibility effects for ON-NO than for OO-NN because the remote flankers and near flankers are associated with opposite responses.

### Method

#### Subjects

We tested another group of 32 subjects sampled from Prolific. We used the same inclusion criteria as Experiment 1 but excluded subjects who had participated in Experiment 1. The mean age of the subjects was 27.88 (*SD* = 4.96). Their gender distribution (male:female:prefer-not-to-say) was 17:15:0. In this experiment, 22 subjects were rejected and replaced for not meeting the criterion of 60% correct in a block.

The number of rejected subjects is anomalous. In 14 published episodic flanker experiments run on Prolific (all with 32 subjects), the mean number of subjects rejected and replaced was 3.86 (Logan et al., 2023b, 2024a, 2024b). The range was 0–15. The experiment in which 15 subjects were rejected assessed the compatibility effect with four to six colors, which are more difficult to remember than letters. Excluding that experiment, the range was 0–7. In five episodic flanker experiments run in person in the laboratory (prepandemic), the mean number of rejected subjects was 2.80 (range: 0–7; Logan et al., 2021).

We determined how far subjects got into the experiment before they were rejected. There were eight blocks of 96 trials. Five subjects were rejected in the first block, six were rejected in the second block, and 11 were rejected in the third block. This suggests they found the task difficult and they would not or could not continue with it. In either case, the rejection rate is anomalous. The data from the subjects we included look much like data from previous episodic flanker experiments with lower rejection rates and the data from Experiment 1 (the OO-NN compatibility effect replicates previous experiments), so we proceeded with the analysis.

#### Apparatus and stimuli

These were almost the same as in Experiment 1, except that the probe displays were constructed differently, as described below.

#### Procedure

The procedure was the same as Experiment 1 except for the construction of the ON-NO context probes. In this experiment, the cued letter in the probe was always surrounded by a single flanker of the same type (both old or both new) in each cue position. The basic design was 4 (Lag − 1, Lag 0, Lag 0, Lag + 1) × 6 (cue positions: 1–6) × 4 (context type: OO, ON, NO, NN), and so required 96 trials. The basic design was replicated eight times to produce 768 trials in total. A different random order was prepared for each subject.

As in Experiment 1, Lag − 1 for Position 1 and Lag + 1 for Position 6 were “reflected” so that Position 1 had two Lag + 1 trials in the basic design and Position 6 had two Lag − 1 trials. OO and NN probes were constructed as before, including all old or all new items in the nontarget positions. ON and NO probes were different. The flankers immediately adjacent to the target (to the left and right of the cued position) were of the same kind, either both old (list: ABCDEF, probe XTCDER) or both new (list: ABCDEF, probe ABNDXF) for each cue position. With this design, the target was always surrounded by flankers of the same kind and the nearest flanker of the opposite kind was always two positions away from the target. Positions 1 and 6 were exceptions. When Position 1 was cued, the two flankers to the right were of the same kind (both old or both new). When Position 6 was cued, the two flankers to the left were always of the same kind. We did this so that targets would always occur in a uniform group of three letters.

#### Data analysis

We assessed the compatibility effect in RT and error rate in OO-NN and ON-NO contexts (separately) with a contrast using weights {− 1 + 1 + 1 − 1} for conditions {same-yes same-no different-yes different-no}. We asked whether compatibility effects differed between OO-NN and ON-NO contexts by comparing their contrast values with another contrast with weights − 1 for the OO-NN contrast and + 1 for the ON-NO contrast. These contrasts focus directly on the theoretically relevant questions. We report an analysis of serial position in the Appendix.

## Results and discussion

**Fig. 3:**
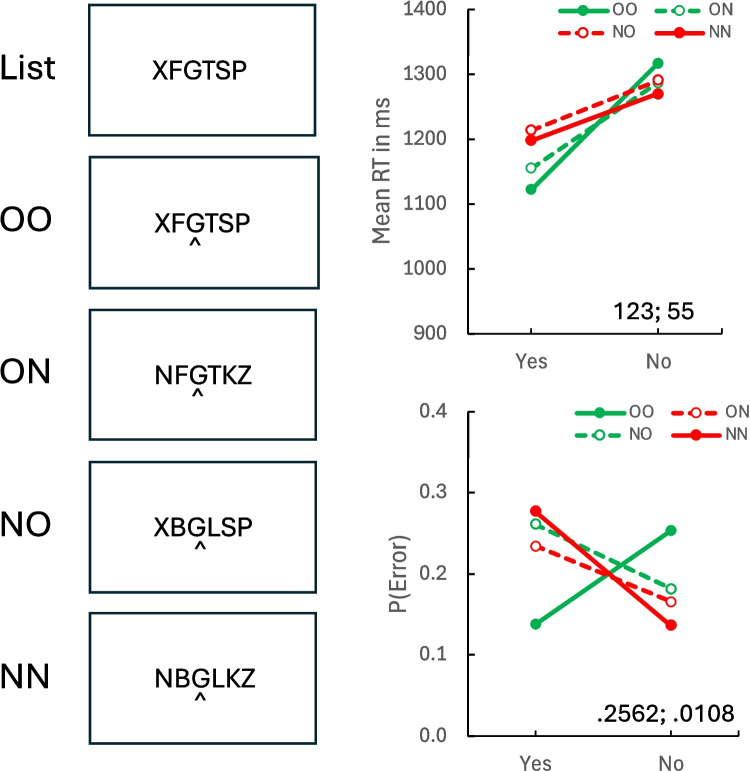
Episodic flanker task list and probes (left column). Mean response time (RT; right column, top panel) and error rate (P(Error); right column, bottom panel) in Experiment 2. OO = old, old (both near and far contexts old); NN = new, new (both near and far contexts new); ON = old, new (near context old, far context new); NO = new, old (near context new, far context old). The values of the compatibility contrasts for OO-NN and ON-NO probes (respectively) are inset in each panel. (Color figure online)

Mean RTs (top right) and error rates (bottom right) are plotted as a function of response (“yes” vs. “no”) and context condition (OO, ON, NO, NN) in Fig. 3. The data for the OO-NN conditions show the crossover pattern typical of the episodic flanker compatibility effect, replicating Experiment 1 and our previous experiments with the same probes. The data for the ON-NO conditions show a weaker crossover pattern for RT and a null interaction for error rate. These results show that remote flankers contribute to the compatibility effect, which is consistent with global matching. The compatibility contrasts, inset in Fig. 2 and reported in Table 2, were significantly greater than zero for both measures in both conditions except for the ON-NO error contrast, which was not significant. The OO-NN compatibility effects were significantly larger than the ON-NO compatibility effects for both RT and error rate, consistent with global matching.

**Table 2:** Contrasts evaluating the episodic compatibility effect in response time and error rate in Experiment 2

*Response time*	*t*(31)	*p*	*SEM*	*N* > 0	*BF*_*10*_
OO-NN	4.1222	.0003	30.5869	27	108.268
ON-NO	2.8448	.0081	19.2194	24	5.4412
OO-NN vs. ON-NO	2.6834	.0019	32.0090	21	3.8900
*Error rate*					
OO-NN	7.8092	<.0001	.0328	31	1,563,066
ON-NO	0.5591	.5804	.0192	19	0.2183
OO-NN vs. ON-NO	5.7709	<.0001	.0425	30	7971.051

OO = old near and far contexts, NN = new near and far contexts, ON = old near and new far contexts, NO = new near and old far contexts. *SEM* = standard error of the mean. *N* > 0 = number of subjects showing a contrast greater than zero. *BF*_*10*_ = Bayes factor supporting the alternative hypothesis

Experiment 2 found clear support for global matching account of the episodic flanker compatibility effect. Far flankers associated with the opposite response diminished the impact of the near flankers and reduced the compatibility effect. Altogether, the data are consistent with a mixture of local matching, which is required to account for overall accuracy (mean hit rate − false alarm rate = 0.5885), and global matching, which is required to account for the effects of the remote flankers.

## Experiment 3

To complete the analogy between attention to memory and attention to perception, we manipulated the compatibility of near and far flankers in a standard B. A. Eriksen and Eriksen (1974) flanker task. To our knowledge, this has not been done before with the perceptual flanker task. On each trial, subjects saw a string of five letters and their task was to press one key if the central letter was an *S* or a *C* and press the other key if the central letter was an *H* or a *K*. The flanking letters were made from combinations of *S*, *C*, *H*, and *K* to produce three kinds of flankers: *Identical flankers* presented the same letter (HHSHH), *same flankers* presented two letters in the same response category (KHSHK), and *different flankers* presented two letters from different response categories (CHSHC). The central target letter was *compatible* with the flankers if it came from the same response category (flankers identical: HHKHH, same: KHKHK, different: CHKHC). It was *incompatible* with the flankers if it came from a different response category (flankers identical: HHSHH, same: KHSHK, different: CHSHC).

The predictions for the perceptual flanker task are the same as the predictions for the episodic flanker task: If local matching is necessary and sufficient, then compatibility effects should only depend on near flankers. The compatibility effects for RT and error rate should be the same for identical, same, and different flankers. If global matching is employed, the compatibility effects should depend on far flankers as well as near flankers. The compatibility effects for RT and error rate should be stronger with identical and same flankers, where near and far flankers point to the same response, than with different flankers, where near and far flankers point to opposite responses.

### Method

#### Subjects

We tested a group of 32 subjects sampled from Prolific, using the same inclusion criteria as Experiments 1 and 2. The mean age of the subjects was 28.69 (*SD* = 6.12). Their gender distribution (male:female:prefer-not-to-say) was 14:17:1. In this experiment, one subject was rejected and replaced for not meeting the criterion of 60% correct in a block.

#### Apparatus and stimuli

 The experiment was run on subjects’ own computers, as in previous experiments. The displays were different. They consisted of strings of five letters, made from the letters* S*, *C*, *H*, and *K*, following B. A. Eriksen and Eriksen’s (1974) original design. The font and spacing was the same as in the previous experiments.

#### Procedure

On each trial, subjects saw a fixation display for 1,000 ms. It was replaced by the string of five letters centered on the fixation point, which remained on until they responded. Then the screen went blank for 1,000 ms until the next trial began. There were 768 trials altogether. The basic design involved the 16 possible pairings of the four letters (allowing letters to repeat). Each letter was presented once as a target with each of the 16 pairs of flankers, producing a set of 64 trials that implement the basic design. These 64 trials were presented 12 times in random order to produce a total of 768 trials for each subject. The three flanker types (identical, same, different) were subsets of the 16 pairings. There 192 trials with identical flankers, 192 trials with same flankers, and 384 trials with different flankers. Compatibility was defined by the response category of the target and its near neighbors. They were in the same response category on compatible trials and different response categories on incompatible trials. Half of the trials in each flanker condition were compatible and half were incompatible.

#### Data analysis

We analyzed the data with planned contrasts that capture the theoretically relevant effects. We calculated a compatibility contrast within each flanker condition, comparing conditions in which the target and near flanker were in the same response category with conditions in which they were in different response categories. We compared the strength of the flanker effects in each pair of conditions. The critical contrasts are between identical and different flanker conditions and between same and different flanker conditions. The latter comparison is most like the comparisons of OO-NN and ON-NO conditions in Experiments 1 and 2.

## Results and discussion

**Fig. 4:**
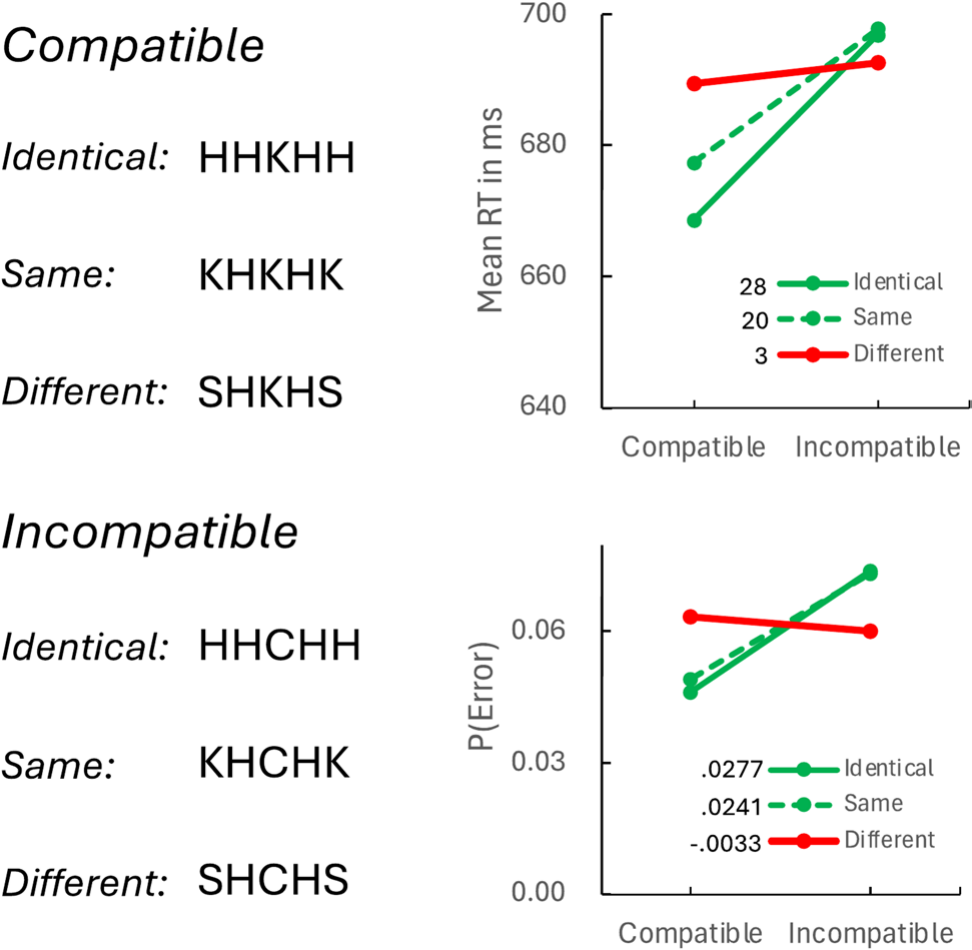
Perceptual flanker task displays (left) and results (right) for Experiment 3. Compatible and incompatible refer to the relation between the central target and the immediately adjacent flankers. Identical, same, and different refer to relations between the flankers. Identical = same letters; Same = letters from the same response category; Different = letters from opposite response categories. The magnitude of the compatibility effect is inset near the legends. (Color figure online)

The mean RTs (top right) and error rates (bottom right) are plotted in Fig. 4. RTs and error rates were lower when adjacent flankers were compatible with the target than when they were incompatible, replicating B. A. Eriksen and Eriksen (1974) and our previous episodic flanker experiments. The compatibility effects were modulated by the nature of the far flankers. They were strong when near and far flankers were identical or in the same response category but they were weak when far and near flankers came from different response categories, conceptually replicating Experiments 1 and 2 and suggesting that the perceptual flanker task depends on a combination of global and local matches, as many theorists have claimed (Cohen et al., 1992; Hübner et al., 2010; White et al., 2011).

Contrasts evaluating the significance of the compatibility contrast in each flanker condition and contrasts comparing conditions are presented in Table 3. The compatibility effects for RT and error rate were significant with identical and same flankers but not with different flankers. The identical flanker effects and the same flanker effects were stronger than the different flanker effects for both measures but not significantly different from each other in either measure.

**Table 3:** Contrasts evaluating the perceptual flanker compatibility effects in response time and error rate in Experiment 3

*Response time*	*t*(31)	*p*	*SEM*	N > 0	*BF*_*10*_
Identical	4.1727	.0002	6.7582	24	122.9382
Same	3.5020	.0014	5.8314	23	23.8107
Different	0.6708	.5073	4.7612	21	0.2350
Identical–Different	3.0412	.0048	8.2224	24	8.3135
Same–Different	3.6184	.0010	4.7612	21	31.4026
Identical–Same	0.9939	.3280	7.8263	18	0.2971
*Error rate*					
Identical	3.1025	.0041	.0089	20	9.5203
Same	3.4716	.0015	.0069	22	23.1650
Different	−0.5509	.5856	.0059	16	0.2173
Identical–Different	2.4859	.0185	.0124	21	2.6247
Same–Different	4.6213	.0001	.0059	16	386.988
Identical–Same	0.4649	.6446	.0077	13	0.2087

The perceptual flanker task showed the same effects of far flankers as the episodic flanker task, strengthening the empirical parallels between the tasks and the broader analogy between attention turned outward inward and outward. In perception and memory, far flankers modulate the effects of near flankers, strengthening them if they point to the same response category and weakening them if they point to opposite response categories. The results suggest that the perceptual flanker effect depends on global and local matches, as theorists have claimed (Cohen et al., 1992; Hübner et al., 2010; White et al., 2011), strengthening theoretical analogy further. Attention to perception and memory is both global and local.

## General discussion

The experiments assessed the role of global matching in the episodic flanker compatibility effect by manipulating the compatibility of contexts near and far from the focus of attention. Global matching predicts a reduction in the compatibility effect when near and far flankers point to opposite responses (ON-NO relative to OO-NN). Local matching predicts no effect of remote flankers and so no reduction in the compatibility effect. The results of the episodic flanker experiments (1 and 2) support global matching. The compatibility effect was smaller with ON-NO probes than with OO-NN probes for error rate in both experiments and for RT for Positions 4–6 (right side) in Experiment 1 and all positions in Experiment 2. The RT contrast for Positions 1–3 (left side) in Experiment 1 was not significant, suggesting local matching or a strong left-to-right bias in memory strength.

Experiment 3 strengthened the analogy between attention turned outward and inward by manipulating near and far flankers in the B. A. Eriksen and Eriksen (1974) perceptual flanker task and finding the same modulatory effects of far flankers consistent with global matching. Attention turned inward and outward addresses structures globally and locally.

The episodic flanker results are consistent with the modeling results in previous experiments that showed better fits when global and local matches were both included and the empirical results that showed reduced compatibility effects for probes with scrambled or swapped letters (Logan et al., 2021). Those experiments provide converging evidence for the same conclusions: Like attention turned outward, attention turned inward can address a whole structure or focus on an element in that structure. Performance in perceptual and episodic flanker tasks suggests that attention can engage both matches at the same time, weighing the information that local and global matches provide (Cohen et al., 1992; Hübner et al., 2010; Ulrich et al., 2015; White et al., 2011; see Eq. 4).

The present results contrast with results from experiments with a single flanker, which suggested that local matches are sufficient to produce the episodic flanker compatibility effect (Logan et al., 2024a, 2024b). A single flanker adjacent to the target produced compatibility effects in RT that were about as large as those from previous experiments (and the present one) with multiple flankers, though the compatibility effects for error rate were smaller (see Logan et al., 2024a, 2024b, Table 4). A single flanker one position away from the target produced a compatibility effect for error rate but not for RT, suggesting a sharp focus of attention on the cued item that is predicted by a local match.

The single flanker results imply that global matching is not necessary to produce the episodic flanker compatibility effect, but they do not imply that global matching is not sufficient. The single flanker experiments removed the items from the probe that would support a global match (i.e., remote items not adjacent to the cued position), so we should expect little evidence of global matching. The present experiments manipulated the compatibility of probe items near and far from the cued position and found evidence of global matching: Remote flankers influenced the compatibility effect. However, the present experiments also found evidence for local matching, in that subjects were able to focus sharply on the cued position and reject lures from adjacent positions. Thus, the best account of the episodic flanker task appears to require a combination of global and local matches.

Local matching is well understood theoretically (Eqs. 1–4). In our thinking, it results from applying a retrieval cue to a memory representation. The retrieval cue is the spotlight of attention that focuses on the cued position in memory. Logan et al. (2021) developed local matching models based on three major theories of serial memory: the perturbation model (Estes, 1972; Lee & Estes, 1981; Ratcliff, 1981), the start–end model (Henson, 1998), and the context retrieval and updating model (Logan, 2021; Lohnas, 2024). Each model explains how items are associated with a structure that represents serial order and how items can be retrieved from that structure with the appropriate cues. Logan et al. (2021) used these representations and retrieval cues to model focused attention in the episodic flanker task, and each model captured the compatibility effects. The best-fitting models included a global match as well as a local match.

The local match is well defined conceptually, computationally, and mathematically in the memory theories and the postulates connecting them to attention. The global match is not well defined conceptually or computationally our models of the episodic flanker task. It was defined mathematically as the sum of local match values between corresponding items in the memory list and the probe (Eq. 3). This definition was sufficient for model fitting, but we did not describe the match conceptually beyond saying it represented the list items in order. Nor did we describe computations required to match items in corresponding positions and combine the results into a global match value. These missing steps are essential in developing a satisfactory explanation of performance in the episodic flanker task and the perceptual flanker task. Understanding these steps is an important goal for future research.

Global matching is well defined conceptually, computationally, and mathematically in models of *item recognition* tasks, in which subjects have to indicate whether a probe item occurred anywhere on a list (Clark & Gronlund, 1996; Osth & Dennis, 2024), but this global matching is different from that in the episodic flanker (position-cued recognition) task. In global matching models of item recognition, a single probe item is compared with the entire memory list without regard to its position in the list. In our model of global matching in position-cued recognition, the entire probe is compared with the entire memory list, and the degree of match is determined by the number of items that appear in the same position in the probe and the list. This requires a representation of order, a process that calculates the match separately at each position, and a process that integrates the matches across positions to generate the global match value. These processes are not well defined. Defining them conceptually and computationally is an important goal for future research.

Global matching is well defined mathematically in models of the perceptual flanker task, but it is not well defined computationally (Cohen et al., 1992; Hübner et al., 2010; White et al., 2011). The models assume all items in the flanker display are processed, but they say nothing about how (or whether) items are bound to positions. They would have trouble predicting the compatibility effects in Experiment 3, which depend on the flankers’ positions relative to the target. If the models were applied to the episodic flanker task, they would not explain the effects of scrambling and swapping letters in the probe (Logan et al., 2021) or the results of Experiments 1 and 2. A deeper explanation would require an account of how items are bound to positions to represent serial order.

The models of the perceptual flanker task have addressed the temporal dynamics of global and local matching. Their methods and models may be adapted profitably to the episodic flanker task. Some models assume that the global match begins before attention is focused on the cued position and the local match contributes later (Cohen et al., 1992) or replaces (Hübner et al., 2010) the global match once attention is focused. White et al. (2011) assume a “shrinking spotlight” that is focused on the whole display at first and then contracts over time until it focuses only on the target (Eriksen & St. James, 1986). Ulrich et al. (2015) assume the effect of the flankers is transient and define its time-course (a gamma function). The flankers contribute briefly and then stop contributing. Our models of the episodic flanker task (Logan et al., 2021) assume simpler dynamics: The contribution from global and local matches begins at the same time and remains constant until a response is made (i.e., the drift rates in diffusion models of the two processes are constant over time; see Eq. 4). It would be interesting to examine the dynamics in the episodic flanker task empirically and theoretically, applying methods and models from the perceptual flanker task. They might reveal important insights into the nature of global and local matching.

Research from the last century on the *same–different* task promises some insight into the nature of the global matching required in the episodic flanker task (see Farell, 1985, for a review). The task requires comparing two strings of objects (usually letters) and distinguishing *same* pairs in which all of the corresponding objects in the two strings are identical (e.g., ABCDEF, ABCDEF) from *different* pairs in which one or more of the objects differ between the strings, by substitution (ABCDEF, ABCXEF) or transposition (ABCDEF, ABDCEF). Much of the research was driven by the *fast same effect*, whereby same RTs were shorter than the most difficult different RTs. This result contradicts a serial comparison model in which “same” responses require exhaustive comparison of each item in the two strings while “different” responses can self-terminate when the first mismatch is detected. The serial model predicts longer “same” RTs, but they are shorter. Global matching was proposed to explain this effect: Same responses are generated by an “identity reporter” that matches the strings as wholes while different responses are generated by a comparison process that looks for differences between corresponding objects (e.g., Bamber, 1969; Farell, 2022). Other explanations proliferated (see Farell, 1985) and formal models offered accounts based on response bias (Ratcliff, 1981; Ratcliff & Hacker, 1981), rechecking mismatches (Krueger, 1978), and priming (Proctor, 1981). The global identity reporter account was not expressed computationally or mathematically and so provides little guidance for our understanding of how global matching is implemented in episodic and perceptual flanker tasks. This, too, motivates further research into the nature of global matching. A recent resurgence of interest in the same–different task may yield important insights (Cousineau et al., 2023; Farell, 2022).

## Conclusions

We asked whether the episodic flanker compatibility effect depends on global matching of probes to memory lists by manipulating the compatibility of near and far flankers independently. Global matching assumes that both near and far flankers contribute to the compatibility effect, so the effect should be stronger when near and far flankers point to the same response than when they point to opposite responses. Local matching predicts no effect of far flankers. The compatibility effect should be the same whether the far flankers point to the same or the opposite response as the near flankers. In two experiments, we found stronger flanker compatibility effects when near and far flankers agreed than when they disagreed, supporting a role for global matching. Local matching is necessary to perform the task accurately, and accuracy was well above chance in both experiments, so we conclude that the episodic flanker effect depends on a combination of global and local matching. Experiment 3 replicated these effects in the standard perceptual flanker task. Our results strengthen the parallels between the episodic and perceptual flanker tasks and provide converging support for the conjecture that memory retrieval is attention turned inward.

## Supplementary Information

Below is the link to the electronic supplementary material.

Supplementary file1 (DOCX 311 KB)

## Data Availability

The data and software are posted on Open Science Framework (https://osf.io/sv8ag/). This includes the raw data, the analyzed data, the analysis programs, and the programs for conducting the experiments online.

## References

[CR1] Anderson, J. R. (1978). Arguments concerning representations for mental imagery. *Psychological Review,**85*(4), 249–277.

[CR2] Bamber, D. (1969). Reaction times and error rates for “same”-“different” judgments of multidimensional stimuli. *Perception & Psychophysics,**6*(3), 169–174.

[CR3] Broadbent, D. E. (1957). A mechanical model for human attention and immediate memory. *Psychological Review,**64*, 205–215. 13441856 10.1037/h0047313

[CR4] Brown, G. D. A., Neath, I., & Chater, N. (2007). A temporal ratio model of memory. *Psychological Review,**111*(3), 539–576. 10.1037/0033-295X.114.3.539 17638496

[CR5] Chun, M. M., Golomb, J. D., & Turk-Browne, N. B. (2011). A taxonomy of external and internal attention. *Annual Review of Psychology,**62*, 73–101. 19575619 10.1146/annurev.psych.093008.100427

[CR6] Clark, S. E., & Gronlund, S. D. (1996). Global matching models of recognition memory: How the models match the data. *Psychonomic Bulletin & Review,**3*, 37–60. 24214802 10.3758/BF03210740

[CR7] Cohen, J. D., Servan-Schreiber, D., & McClelland, J. L. (1992). A parallel distributed processing approach to automaticity. *The American Journal of Psychology,**105*(2), 239–269. 1621882

[CR8] Cousineau, D., Harding, B., Walker, J. A., Durand, G., & T. -Groulx, J., Lauzon, S., & Goulet, M.-A. (2023). Analyses of response time data in the same–different task. *Canadian Journal of Experimental Psychology/revue Canadienne De Psychologie Expérimentale,**77*(2), 115–129. 37036687 10.1037/cep0000301

[CR9] Cowan, N. (1988). Evolving conceptions of memory storage, selective attention, and their mutual constraints within the human information-processing system. *Psychological Bulletin,**104*(2), 163–191. 3054993 10.1037/0033-2909.104.2.163

[CR10] Craik, F. I. M., & Tulving, E. (1975). Depth of processing and the retention of words in episodic memory. *Journal of Experimental Psychology: General,**104*(3), 268–294.

[CR11] de Leeuw, J. R. (2015). jsPsych: A JavaScript library for creating behavioral experiments in a Web browser. *Behavior Research Methods,**47*, 1–12. 24683129 10.3758/s13428-014-0458-y

[CR12] Eriksen, B. A., & Eriksen, C. W. (1974). Effects of noise letters upon the identification of a target letter in a nonsearch task. *Perception & Psychophysics,**16*(1), 143–149.

[CR13] Eriksen, C. W., & St. James, J. D. (1986). Visual attention within and around the field of focal attention: A zoom lens model. *Perception & Psychophysics,**40*(4), 225–240. 3786090 10.3758/bf03211502

[CR14] Estes, W. K. (1972). An associative basis for coding and organization in memory. In A. W. Melton & E. Martin (Eds.), *Coding processes in human memory* (pp. 161–190). Winston.

[CR15] Farell, B. (1985). “Same”-“different” judgments: A review of current controversies in perceptual comparisons. *Psychological Bulletin,**98*(3), 419–456. 4080894

[CR16] Farell, B. (2022). Hypothesis testing, attention, and ‘same’-‘different’ judgments. *Cognitive Psychology, 132,* Article 101443. 10.1016/j.cogpsych.2021.101443 34856532

[CR17] Fischer-Baum, S., & McCloskey, M. (2015). Representation of item position in immediate serial recall: Evidence from intrusion errors. *Journal of Experimental Psychology: Learning, Memory, and Cognition,**41*(5), 1426–1446. 25730307 10.1037/xlm0000102

[CR18] Henson, R. N. (1998). Short-term memory for serial order: The start-end model. *Cognitive Psychology,**36*(2), 73–137. 9721198 10.1006/cogp.1998.0685

[CR19] Hübner, R., Steinhauser, M., & Lehle, C. (2010). A dual-stage two-phase model of selective attention. *Psychological Review,**117*(3), 759–784. 20658852 10.1037/a0019471

[CR20] James, W. (1890). *The principles of psychology*. Holt.

[CR21] Krueger, L. E. (1978). A theory of perceptual matching. *Psychological Review,**85*(4), 278–304. 684134

[CR22] Lee, C. L., & Estes, W. K. (1981). Item and order information in short-term memory: Evidence for multilevel perturbation processes. *Journal of Experimental Psychology: Human Learning and Memory,**7*(3), 149–169.

[CR23] Lewandowsky, S., & Farrell, S. (2008). Short-term memory: New data and a model. *Psychology of Learning and Motivation,**49*, 1–48.

[CR24] Logan, G. D. (2021). Serial order in perception, cognition, and action. *Psychological Review,**128*, 1–44. 32804525 10.1037/rev0000253

[CR25] Logan, G. D., Afu, K. C. S., Haynes, B. E., Weeks, E. E., Ulrich, J. E., & Lilburn, S. D. (2024a). Attention focused on memory: The episodic flanker effect with letters, words, colors, and pictures. *Attention, Perception & Psychophysics,**86*, 2690–2706. 10.3758/s13414-024-02965-9 PMC11652678 39384681

[CR26] Logan, G. D., & Cox, G. E. (2021). Serial memory: Putting chains and position codes in context. *Psychological Review,**128*, 1197–1205. 34570522 10.1037/rev0000327

[CR27] Logan, G. D., Cox, G. E., Annis, J., & Lindsey, D. R. B. (2021). The episodic flanker effect: Memory retrieval as attention turned inward. *Psychological Review,**128*, 397–445. 33939456 10.1037/rev0000272

[CR28] Logan, G. D., Lilburn, S. D., & Ulrich, J. E. (2023a). Serial attention to serial memory: The psychological refractory period in forward and backward cued recall. *Cognitive Psychology, 145*, Article 101583. 10.1016/j.cogpsych.2023.101583 37429216

[CR29] Logan, G. D., Lilburn, S. D., & Ulrich, J. E. (2023b). The spotlight turned inward: The time-course of focusing attention on memory. *Psychonomic Bulletin & Review,**30*, 1028–1040. 36471229 10.3758/s13423-022-02222-w

[CR30] Logan, G. D., Lindsey, D. R. B., & Ulrich, J. A. (2024b). The power of one: A single flanker produces compatibility effects in the episodic flanker task. *Memory & Cognition, Advance Online Publication.*10.3758/s13421-024-01653-1 10.3758/s13421-024-01653-1 PMC12307454 39470959

[CR31] Lohnas, L. J. (2024). A retrieved context model of serial recall and free recall. *Computational Brain & Behavior. Advance Online Publication.*10.1007/s42113-024-00221-9 10.1007/s42113-024-00221-9 PMC13298665 42459226

[CR32] Mewhort, D. J. K., Merikle, P. M., & Bryden, M. P. (1969). On the transfer from iconic to short-term memory. *Journal of Experimental Psychology,**81*(1), 89–94.

[CR33] Nobre, A. C., Coull, J. T., Maquet, P., Frith, C. D., Vandernberghe, R., & Mesulam, M. M. (2004). Orienting attention to locations in perceptual versus mental representations. *Journal of Cognitive Neuroscience,**16*, 363–373. 15072672 10.1162/089892904322926700

[CR34] Norman, D. A. (1968). Toward a theory of memory and attention. *Psychological Review,**75*, 522–536.

[CR35] Osth, A. F., & Dennis, S. (2024). Global matching models of recognition memory. *The Oxford handbook of human memory, two volume pack: Foundations and applications* (pp. 895–922). Oxford University Press.

[CR36] Proctor, R. W. (1981). A unified theory for matching-task phenomena. *Psychological Review,**88*, 291–326. 6844477

[CR37] Ratcliff, R. (1981). A theory of order relations in perceptual matching. *Psychological Review,**88*(6), 552–572.

[CR38] Ratcliff, R., & Hacker, M. J. (1981). Speed and accuracy of same and different responses in perceptual matching. *Perception & Psychophysics,**30*(3), 303–307. 7322806 10.3758/bf03214286

[CR39] Rugg, M. D., Johnson, J. D., Park, H., & Uncapher, M. R. (2008). Encoding-retrieval overlap in human episodic memory: A functional neuroimaging perspective. In W. S. Sossin, J.-C. Lacaille, V. F. Castellucci, & S. Belleville (Eds.). *Progress in brain research* (Vol. 169, pp. 339–352). Elsevier. 10.1016/S0079-6123(07)00021-0 18394485

[CR40] Tulving, E. (1972). Episodic and semantic memory. In E. Tulving & W. Donaldson (Eds.), *Organization and memory* (pp. 381–402). Academic Press.

[CR41] Ulrich, R., Schröter, H., Leuthold, H., & Birngruber, T. (2015). Automatic and controlled stimulus processing in conflict tasks: Superimposed diffusion processes and delta functions. *Cognitive Psychology,**78*, 148–174. 25909766 10.1016/j.cogpsych.2015.02.005

[CR42] White, C. N., Ratcliff, R., & Starns, J. J. (2011). Diffusion models of the flanker task: Discrete versus gradual attentional selection. *Cognitive Psychology,**63*(4), 210–238. 21964663 10.1016/j.cogpsych.2011.08.001 PMC3195995

[CR43] Woodman, G. F., Wang, S., Sutterer, D. W., Reinhart, R. M. G., & Fukuda, K. (2022). Alpha suppression indexes a spotlight of visual-spatial attention that can shine on both perceptual and memory representations. *Psychonomic Bulletin & Review,**28*, 681–698. 10.3758/s13423-021-02034-4 PMC10067153 34877635

