# Supplementary material for: The power of many: The role of global matching in the episodic flanker compatibility effect
Source: Mem Cognit. 2025 May 30;53(8):2700–14. doi: 10.3758/s13421-025-01733-w (PMC12696052; doi:10.3758/s13421-025-01733-w)
Supplement: Supplementary file 1 — Supplementary file1 (DOCX 311 KB) [file 13421_2025_1733_MOESM1_ESM.docx]

**The Power of Many:**

**The Role of Global Matching in the Episodic Flanker Compatibility Effect**

Gordon D. Logan

Simon D. Lilburn

Vanderbilt University

**Appendix: Serial Position Effects in Experiments 1 and 2**

The analyses in Experiments 1 and 2 collapsed over serial position to increase the number of observations, resulting in a total of 96 observations in each combination of conditions (OO, NN, ON, NO) and responses (“yes,” “no”). Previous episodic flanker experiments show serial position effects in RT and error rate. Response time increases for the first few positions, peaks, and decreases for the last position. Error rate changes similarly. We have assumed the flanker compatibility effects are the same at each serial position to justify collapsing over serial position. Here, we test that assumption.

The compatibility effects in RT (top) and error rate (bottom) are plotted as a function of serial position in Figure A1 for Experiment 1 and Figure A2 for Experiment 2. Each point in the figure is based on only 16 observations per subject, so the data are much noisier than the data in Figures 2 and 3. The values of the compatibility contrasts for each serial position are inset in the figures. Asterisks indicate which ones are significant at *p* < .05. In the OO-NN conditions, the contrasts were significant for RT and error rate at each serial position in both experiments. In the ON-NO, the contrasts were significant at two positions for RT and three positions in error rate in Experiment 1and at two positions for RT and one position for error rate in Experiment 2.

**Figure A1: Serial Position Effects in Experiment 1**

**Figure A1 caption:** Mean response time (RT; top panels) and error rate (P(Error); bottom panels) in Experiment 1 as a function of compatibility (same-different x yes-no) and serial position. OO = old, old (both near and far contexts old); NN = new, new (both near and far contexts new); ON = old, new (near context old, far context new); NO = new, old (near context new, far context old). The values of the compatibility contrasts for OO-NN and ON-NO probes in each serial position are inset in each panel. * = *p* < .05.

**Figure A2: Serial Position Effects in Experiment 2**

**Figure A2 caption:** Mean response time (RT; top panels) and error rate (P(Error); bottom panels) in Experiment 2 as a function of compatibility (same-different x yes-no) and serial position. OO = old, old (both near and far contexts old); NN = new, new (both near and far contexts new); ON = old, new (near context old, far context new); NO = new, old (near context new, far context old). The values of the compatibility contrasts for OO-NN and ON-NO probes in each serial position are inset in each panel. * = *p* < .05.

We conducted 2 (condition: OO-NN vs. ON-NO) x 6 (serial position) ANOVAs on the contrast values in RT and error rate to assess the significance of the apparent differences in Figures A1 and A2. Summary tables for the ANOVAs are presented in Table A1. In the RT ANOVA in Experiment 1, condition was the only significant effect. In the error rate ANOVA in Experiment 1, the main effects of condition and serial position were significant, but the interaction between them was not. In the RT ANOVA in Experiment 2, no effects were significant. In the error rate ANOVA in Experiment 2, the main effect of condition, the main effect of serial position, and the interaction between them were significant.

The ANOVAs suggest that the RT compatibility effects were not affected by serial position. The error rate results varied with serial position in both experiments, and the condition effect varied with serial position in Experiment 2 but the contrast was greater than zero in the OO-NN condition at all serial positions (see insets in Figure A2). These results should be interpreted with caution because there are so few observations per subject. Taken at face value, they suggest that serial position effects may be interesting to investigate but they did not contradict the results of the main analyses that collapsed over serial position.

**Table A1**

Analyses of variance on compatibility contrast values in RT and error rate as a function of condition (OO-NN vs. ON-NO) and serial position (1-6) in Experiments 1 and 2.

| Effect | F | df | p | MSerror | $\eta_{p}^{2}$ |
| --- | --- | --- | --- | --- | --- |
| Experiment 1 Response Time | | | | | |
| Condition (C) | 12.2471 | 1,31 | .0014 | 76840.8749 | .0429 |
| Serial Position (S) | 0.8704 | 5,155 | .5025 | 54923.8962 | .0109 |
| C x S | 1.8804 | 5,155 | .1008 | 60087.1661 | .0257 |
| Experiment 1 Error Rate | | | | | |
| Condition (C) | 25.2440 | 1,31 | <.0001 | .1692 | .1576 |
| Serial Position (S) | 5.9784 | 5,155 | <.0001 | .0475 | .0524 |
| C x S | 0.6301 | 5,155 | .6770 | .0557 | .0065 |
| Experiment 2 Response Time | | | | | |
| Condition (C) | 3.6713 | 1,31 | .0656 | 78670.0023 | .0133 |
| Serial Position (S) | 1.9863 | 5,155 | .0836 | 51477.6483 | .0235 |
| C x S | 1.9066 | 5,155 | .0962 | 63948.9648 | .0280 |
| Experiment 2 Error Rate | | | | | |
| Condition (C) | 35.5234 | 1,31 | <.0001 | .1628 | .1898 |
| Serial Position (S) | 3.1849 | 5,155 | .0091 | .0451 | .0236 |
| C x S | 2.7915 | 5,155 | .0192 | .0706 | .0324 |
